# Greenness and its interaction with air pollution in relation to postmenopausal breast cancer risk in UK Biobank

**DOI:** 10.1371/journal.pone.0334744

**Published:** 2025-11-12

**Authors:** Carmen Smotherman, Brian L. Sprague, Dejana Braithwaite, Lusine Yaghjyan

**Affiliations:** 1 Department of Epidemiology, College of Public Health and Health Professions and College of Medicine, University of Florida, Gainesville, Florida, United States of America; 2 Department of Surgery, University of Vermont, Burlington, Vermont, United States of America; University of Ferrara, ITALY

## Abstract

It is unclear if natural vegetation (greenness) is associated with breast cancer breast cancer risk and if it interacts with air pollution. We investigated the associations of greenness with breast cancer risk in postmenopausal women, and the extent to which the association of the air pollutant PM_10_ with breast cancer differs by greenness level. Of the 154,804 postmenopausal women included in the study, 6,131 developed breast cancer after the baseline assessment for enrollment into the UK Biobank. Data on greenness measures, and established breast cancer risk factors were available at baseline assessment (2006−2010), while data for PM_10_ were available from 2007 and 2010. We used Cox proportional hazards models to assess adjusted associations between greenness and breast cancer risk. We also examined associations of PM_10_ with breast cancer risk by greenness levels and their interactions. For each 0.1 unit increase in NDVI, we found a 2.6% increase in breast cancer risk (Hazard ratio [HR]=1.03, 95%CI 1.00–1.05). We also found evidence of a significant interaction, with stronger association between cumulative average PM_10_ and breast cancer risk at lower NDVI levels (1^^st^^ quartile NDVI: HR per 10 µg/m^^3^^ PM_10_ = 3.03, 95%CI 1.98–4.64) versus higher NDVI levels (4^^th^^ quartile: HR = 1.52, 95%CI 1.16–1.98). We did not find interactions with other greenness measures. Our results suggest that various measures of greenness differ in their association with breast cancer and that there may be an interaction between greenness and PM_10_ exposure in relation to breast cancer risk.

## Introduction

Natural green environments (greenness), commonly assessed using remote sensing indices such as the Normalized Difference Vegetation Index (NDVI), may reduce harmful environmental exposures (such as air pollution, extreme heat), increase physical activity and reduce stress levels, which subsequently could impact health outcomes [1–5]. Importantly, use of greenness in epidemiologic studies captures multiple interrelated effects rather than isolating individual health benefits. Multiple lines of evidence suggest that greenness improves health, as well as social and environmental outcomes especially in populations with lower socioeconomic status [6]. Previous studies have linked exposure to urban greenness with various health endpoints [7–13], demonstrating in some instances interactions with air pollution [14–16], and in others, an independent effect [17,18]. However, the associations of greenness with cancer risk remain poorly understood. A recent report from Nurses’ Health Study found an inverse association of greenness with overall cancer mortality, independent of air pollution [19]. Two studies suggested an inverse association between greenness and prostate cancer risk, independently of air pollution, social and demographic characteristics, and lifestyle factors [20,21]. A few recent reports found an inverse association of greenness with breast cancer risk [20,22,23], while other reported a positive association [24]. These contrasting findings may be due to differences in study designs, geographic setting, exposure assessment methods, and population characteristics. Some studies suggested that these associations were independent of air pollution, physical activity, and obesity [22,24]. Recent studies also demonstrated associations of greenness with several biomarkers implicated in breast carcinogenesis, including lower insulin resistance and less obesity [2,25,26], lower levels of pro-inflammatory markers [27,28], improved cortisol profiles [12], and longer telomeres [29], thus further supporting a potential link to breast carcinogenesis.

For development and implementation of efficient public health interventions aimed to reduce breast cancer incidence, the potential complex relationship between greenness and air pollution in relation to breast cancer should be comprehensively explored to determine if greenness is indeed associated with breast cancer risk and whether the association is independent of air pollution [30]. Failing to account for the confounding or modifying effects of greenness could misrepresent the true impact of air pollution on health. Understanding these independent and joint effects is critical for public health and urban planning, as it allows for more targeted interventions, e.g., designing green spaces that maximize health benefits while minimizing harmful exposures. It also helps in identifying population subgroups who may benefit most from greenness or be most vulnerable to pollution.

The goal of this study was to examine the association of greenness with postmenopausal breast cancer risk, as well as explore whether association of air pollution with breast cancer differs by the level of greenness for postmenopausal women enrolled in the UK Biobank. We focused on pollution measures that were previously linked to breast cancer risk in our analyses using data from 155,235 postmenopausal women from UK Biobank, specifically PM_10_ annual averages from 2007 and cumulative average PM_10_ (calculated as the average across measures from 2007 and 2010) [31].

## Methods

### Study population

This study included postmenopausal women selected from participants in UK Biobank. The data received from the UK Biobank were accessed for research purposes on January 20, 2021. The authors did not have access to information that could identify individual participants during or after data collection. The recruitment, enrollment and follow-up processes were described in details elsewhere [32]. In short, about 500,000 volunteers, 40–69 years old from the UK were recruited during 2006–2010, and about 20% of the participants completed a follow-up first assessment during 2012–2013.

**Fig 1:**
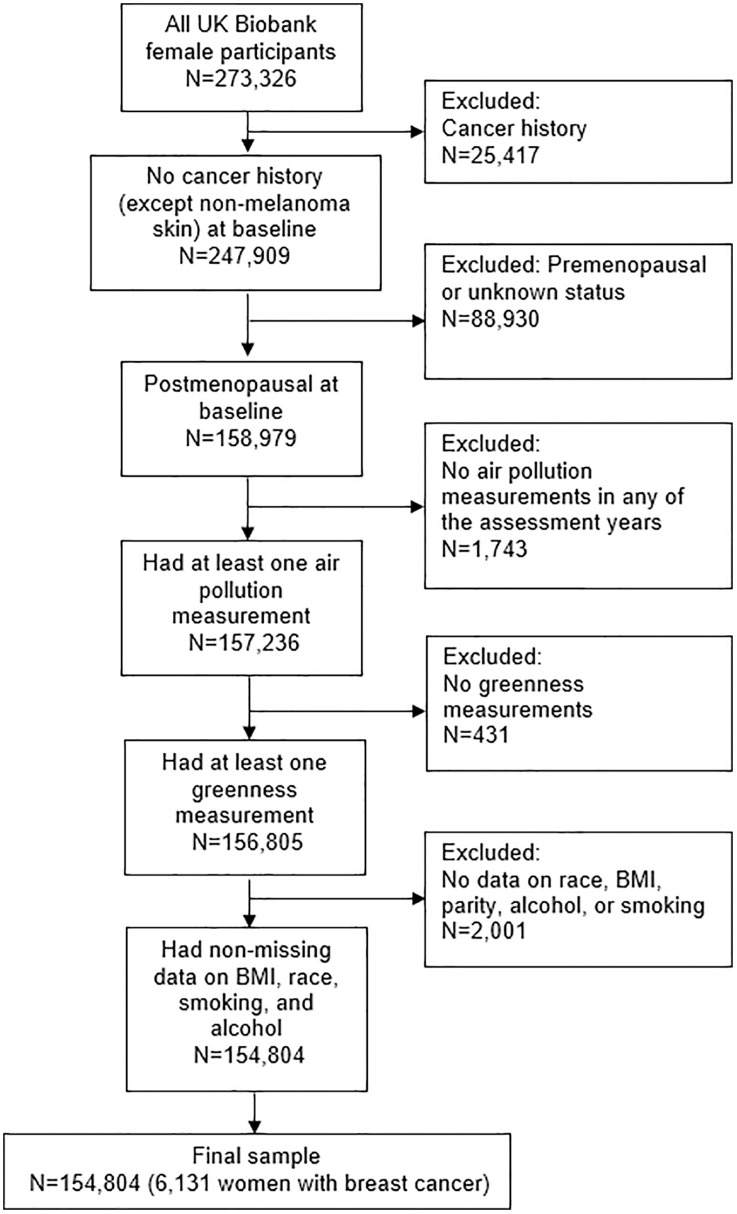
Study sample selection diagram. Flow diagram illustrating the selection process of participants for the study.

In this study, we used the same subset of postmenopausal women from our previous study [31]. Briefly, exclusion criteria encompassed a history of any cancer except non-melanoma skin cancer, premenopausal or unknown menopausal status, and missing data on all air pollution measures, all greenness measures, and on any of the following covariates: race, BMI, parity, alcohol, and smoking. National Health Service (NHS) Central Registers records from England (through February 29, 2020) and from Scotland (through August 31, 2021) were used to assess participants’ outcomes. The International Classification of Diseases (ICD) codes used to identify a breast cancer diagnosis were 174 and C50 for invasive breast cancer, and 2330 and D05 for in-situ breast cancer. This study included 154,804 women (representing 97% of all eligible women in UK Biobank) and 6,131 developed breast cancer during the follow (Fig 1).

UK Biobank was approved by the North West Multi-centre Research Ethics Committee (MREC), which covers the UK. All participants of UK Biobank provided written consent at recruitment. The current analyses were approved by the University of Florida Institutional Review Board (protocol #202002317).

### Greenness assessment

The following greenness measures were assessed in UK Biobank: Normalized Difference Vegetation Index (NDVI), Natural Environment Percentage (NEP), and Greenspace Percentage (GP). All measures were recorded during the assessment period at baseline (2006–2010), while GP and NEP were also available at the first repeat assessment (2012–2013).

Greenspace percentage was modelled using 2005 data from the Generalized Land Use Database (GLUD) for England, which shows the areas of land use at lower-layer super output areas (LSOAs) for 2001 Census Output Areas in England [33]. GP was estimated as the percentage of LSOAs covered by green space, including gardens. Each residential address was allocated circular distance buffers of 300m and 1000m, respectively. An area-weighted mean of GP coverage was allocated to participants from England, at the baseline and at first repeat assessment. GLUD data did not include addresses from outside of England; therefore, greenness measurements were not assigned by UK Biobank for addresses outside England, resulting in missing data for these participants. GP measurements from baseline assessment and the first repeat assessment were highly correlated (correlation coefficient r = 0.971 [p < 0.001, n = 6,417] for GP, buffer 1000m, and r = 0.968 [p < 0.001, n = 6,417] for GP, buffer 300m); thus, only baseline greenness measurements were used in the analyses.

Natural Environment Percentage (NEP) was derived using the Centre for Ecology and Hydrology (CEH) Land Cover Map (LCM) 2007 data to extend coverage to Wales and Scotland [34].The percentage coverage of “natural environment” were calculated for two buffer sizes (300m and 1000m) around the participant residential address. Only baseline NEP measurements were used in analyses since NEP values from baseline and the first repeat assessment were highly correlated: r = 0.969 (p < 0.001, n = 6,417) for NEP, buffer 1000m, and r = 0.965 (p < 0.001, n = 6,417) for NEP, buffer 300m.

The Normalized Difference Vegetation Index (NDVI) is a unit-less index that indicates a relative overall green healthy vegetation, described previously in details [35]. Residential greenness was expressed as mean deviation in the NDVI values within 500m radial area of the participant home address. The NDVI ranges from −1 (indicating water) to +1 (indicating dense green vegetation). Values of 0.2–0.3 indicate shrub and grassland, while 0–0.1 indicate barren rock, sand, or snow. NDVI measure was not available for participants from northern England or Scotland (total of 45,851 or 28.8% of eligible women).

### Air pollution assessment

Exposures to air pollution were assessed using annual averages of particulate matter PM_10_ derived from Land Use Regression (LUR) models, described in details elsewhere [36–38]. Briefly, the estimates for 2007 were based on PM_10_ concentrations obtained from EuroAirnet, the regulatory air pollution monitoring network in Europe [36]. The predictors in the final model were Satellite-derived surface PM_2.5_ from 2001–2006, the Y coordinate of participant location, length of major (200–2500 meters) and minor (200 meters) roads, altitude, tree canopy (100 meters). The model for 2007 explains about 47% of the variation in measured concentrations (about 50% of the variation in log-transformed PM_10_). The estimates for 2010 were based on the monitoring done by the European Study of Cohorts for Air Pollution Effects (ESCAPE), including two sites in UK, Manchester and London/Oxford [37,38]. Predictors in the best model to predict the annual average of PM_10_ for 2010 differed by region. For Manchester, the final model included surface area of urban green (100 meters), total heavy-duty traffic load of major roads (1000 meters), surface area of semi-natural and forested (300 meters), surface area of high-density residential area (100 meters). The model for London/Oxford included inverse distance and inverse squared distance to the nearest major road, and heavy-duty traffic intensity on nearest major road. These regional models were developed and applied separately to account for differences in urban structure and emission patterns; hence for differences in availability of comparable predictor variables across all regions; a single harmonized national model was not used. Due to poor performance of the model for further locations from the initial ESCAPE study area, participants living beyond 400 km from Greater London (northern England and Scotland), were not assigned PM_10_ concentrations for 2010 to prevent exposure misclassification (n = 11,499 or 7.4% or the study sample).

### Covariates information

Data on established breast cancer risk factors (age at menarche, age at menopause, Body Mass Index (BMI), race/ethnicity, postmenopausal hormone therapy, parity, age at first birth, smoking status, alcohol use, and family history for breast cancer were available from baseline and, for a subset of women, from follow-up assessments. We included only baseline risk factor data in the analyses as we previously found high correlations between baseline and repeat assessments for these risk factors, as reported previously [31].

### Statistical analyses

Associations between each greenness measure and breast cancer risk were assessed using Cox proportional hazards regression models, as previously described [39,40]. Multivariate models also included age at recruitment (continuous), age at menarche (continuous), age at menopause (<46, 46 to <50, 50 to <55, 55 or more years, unknown), BMI (kg/m^2^, continuous), race (Caucasian vs Other races), combined parity and age at first birth (nulliparous, parous and age at first birth 25 years or less, parous and age at first birth more than 25 years, parous and unknown age at first birth), postmenopausal hormone use, family history of breast cancer in first degree relatives, smoking and alcohol consumption. “Other races” included: Mixed (White and Black Caribbean, White and Black African, White and Asian, Any other Mixed background), Asian or Asian British (Indian, Pakistani, Bangladeshi, Any other Asian background), Black or Black British (Caribbean, African, Any other Black background, Chinese or Other ethnic group. Follow-up started at enrollment and ended at the time of breast cancer diagnosis, another cancer type diagnosis, death, or at the last linkage to the cancer registries. Greenness and air pollution measures were modeled as continuous variables and as quartiles, respectively. Risk estimates were described using hazard ratios (HRs) and their 95% confidence intervals (CIs). The regression models included the interaction term between age at recruitment and time due to the fact that age at recruitment violated the proportional hazards assumption [40]. Tests for trend were performed, modeling each greenness measure as an ordinal variable, and using the median greenness level in each category.

The regression models were developed for 2007 PM_10_, 2010 PM_10_, and cumulative average PM_10_ (calculated as the average across available PM_10_ measures) and included the interaction between greenness and PM_10_ (modeled as continuous per 10 µg/m^3^ and as quartiles). The interaction was tested in two ways: with greenness and PM_10_ as continuous exposure, and using the medians for each quartile. Next, the associations between PM_10_ and breast cancer risk were examined across the greenness levels. The analyses of 2007 PM_10_ exposure and 2010 PM_10_ exposure, respectively, had a follow-up time starting with the respective year in which the PM_10_ was available. For analyses of cumulative average exposure to PM_10_, the follow-up start time was defined as the latest available PM_10_ measure.

Additional analyses were performed to (1) allow a 2-year lag in air pollution as previously described [31] and (2) include only diagnoses of invasive breast cancer. In the lagged analysis, we first applied the lag to the individual annual PM₁₀ exposure values and then computed the cumulative PM₁₀ exposure using these lagged values. Significance of effects was assessed at 0.05 level. Statistical analyses were done using SAS (SAS Institute Inc. version 9.4).

## Results

Of the 154,804 postmenopausal women included in the analytical sample, 6,131 (4%) were diagnosed with breast cancer during follow-up. The women included in the study were, on average, 60.1 years old (ranging from 40 to 71 years). Those who developed breast cancer were followed-up for 5.4 years, on average (standard deviation [SD]=3.2 years), while cancer-free women were followed for 10.7 years (SD = 2.0 years). Table 1 presents the distributions of patients’ characteristics at baseline by cancer diagnosis, and by greenspace percentage, buffer 1000m, respectively. As compared to women without breast cancer, women with breast cancer were on average older (60.6 vs 60.1 years, p for difference < 0.0001), had higher BMI (27.6 vs 27.2 kg/m^2^, p for difference < 0.0001), had higher proportion of nulliparous (70.1% vs 16.2%) or lower proportion of having a child before 25 years of age (38.1% vs 40.1% p = 0.001), were more likely to have reported currently using postmenopausal hormone therapy at enrollment (11.6% vs. 8.9%, p < 0.001), and having a family history of breast cancer (11.1% vs. 7.2%, p < 0.001) (Table 1).

**Table 1:** Characteristics of study participants at baseline, by the quartiles of greenspace percentage, buffer 1000m and by breast cancer status.

Characteristic	Greenspace quartiles				Breast cancer status	
	Q1: ≤ 27.94 (N = 34,183)	Q2: < 27.94 - ≤ 42.54 (N = 34,183)	Q3: < 42.54 - ≤ 60.91 (N = 34,183)	Q4: > 60.91 (N = 34,186)	Breast cancer (N = 6,131)	No Breast cancer (N = 148,673)
**Mean (SD)**						
Age at enrollment, years	*59.78 (5.65)*	*60.10 (5.57)*	*60.25 (5.47)*	*60.34 (5.37)*	*60.60 (5.10)*	*60.06 (5.54)*
Follow-up time, years	*10.16 (2.11))*	*10.33 (2.22)*	*10.41 (2.18))*	*10.39 (2.16))*	*5.35 (3.14)*	*10.73 (2.00)*
Age at menarche, years	**12.98 (1.61)**	**12.98 (1.61)**	**12.95 (1.60)**	**12.94 (1.57)**	**12.93 (1.61)**	**12.96 (1.59)**
Age at menopause, years	*49.73 (5.00)*	*49.66 (5.19)*	*49.75 (5.14)*	*49.93 (5.05)*	*50.34 (4.92)*	*49.72 (5.09)*
BMI, kg/m^2^	*27.17 (5.28)*	*27.33 (5.15)*	*27.26 (5.00)*	*26.82 (4.78)*	*27.61 (5.01)*	*27.15 (5.07)*
PM_10_ 2007	*24.78 (2.83)*	*22.90 (2.14)*	*25.51 (1.73)*	*20.12 (1.69)*	*22.09 (2.78)*	*22.00 (2.86)*
PM_10_ 2010	*16.88 (1.50)*	*16.57 (1.60)*	*16.18 (1.75)*	*15.16 (2.11)*	16.21 (1.90)	16.20 (1.89)
**N (%)**						
Race						
White	*30,559 (89.40)*	*32,528 (95.16)*	*33,346 (97.55)*	*33,833 (98.97)*	**5,914 (96.46)**	**142,144 (95.61)**
Other	*3,624 (10.60)*	*1,655 (4.84)*	*837 (2.45)*	*353 (1.03)*	**217 (3.54)**	**6,529 (4.39)**
Parity/AFB, years						
Nulliparous	*5,793 (14.96)*	*5,357 (13.85)*	*5,655 (14.63)*	*8,285 (21.41)*	**1,046 (17.06)**	**24,148 (16.24)**
Any children with AFB ≤ 25 years	*15,232 (39.33)*	*16,504 (42.65)*	*16,466 (42.59)*	*13,807 (35.68)*	**2,335 (38.09)**	**59,616 (40.1)**
Any children with AFB > 25 years	*13,155 (33.87)*	*12,064 (31.18)*	*11,654 (30.14)*	*11,263 (29.11)*	**1,898 (30.96)**	**46,158 (31.05)**
Any children with unknown AFB	*4,583 (11.84)*	*4,767 (12.32)*	*4,890 (12.65)*	*5,342 (13.80)*	**852 (13.9)**	**18,751 (12.61)**
Postmenopausal hormone therapy						
Never used hormones	*17,963 (52.55)*	*17,034 (49.83)*	*16,656 (48.73)*	*16,660 (48.73)*	*2,921 (50.13)*	*74,413 (52.63)*
Past	*11,285 (33.01)*	*12,292 (35.96)*	*12,772 (37.36)*	*12,807 (37.46)*	*2,194 (37.65)*	*53,763 (38.03)*
Current	*3,272 (9.57)*	*3,095 (9.05)*	*2,985 (8.73)*	*3,076 (9)*	*712 (12.21)*	*13,203 (9.34)*
Family history						
Breast cancer	2,482 (7.48)	2,474 (7.44)	2,560 (7.71)	2,641 (7.92)	*681 (11.41)*	*10,750 (7.43)*
No breast cancer	30,692 (92.52)	30,776 (92.56)	30,645 (92.29)	30,703 (92.08)	*5,287 (88.59)*	*133,867 (92.57)*
Smoking status						
Never	*19,086 (55.83)*	*19,702 (57.64)*	*20,375 (59.61)*	*21,004 (61.44)*	**3,483 (56.81)**	**87,210 (58.66)**
Past	*11,831 (34.61)*	*11,602 (33.94)*	*11,317 (33.11)*	*11,147 (32.61)*	**2,123 (34.63)**	**49,585 (33.35)**
Current	*3,266 (9.55)*	*2,879 (8.42)*	*2,491 (7.29)*	*2,035 (5.95)*	**525 (8.56)**	**11,878 (7.99)**
Alcohol intake						
Non-drinker	*2,597 (7.60)*	*2,291 (6.70)*	*1,963 (5.74)*	*1,591 (4.65)*	348 (5.68)	9,123 (6.14)
Past drinker	*1,455 (4.26)*	*1,344 (3.93)*	*1,292 (3.78)*	*1,052 (3.08)*	212 (3.46)	5,604 (3.77)
Current drinker	*30,131 (88.15)*	*30,548 (89.37)*	*30,928 (90.48)*	*31,543 (92.27)*	5,571 (90.87)	133,946 (90.09)

**Abbreviations**: AFB – age at first birth; BMI – Body Mass Index; Q – quartiles; SD – standard deviation.

Note: statistically significant differences at 0.001 level across greenness levels or by breast cancer status presented as italic; statistically significant differences across greenness levels or by breast cancer status at 0.05 level presented as bold.

The average greenness indicators were 45.68% (range 4.96–99.18%) for greenspace percentage, buffer 1000m; 35.77% (range 0.23–99.18%) for greenspace percentage, buffer 300m; 41.46% (range 0–100%) for natural environment percentage, buffer 1000m; 26.72% (range 0–100%) for natural environment percentage, buffer 300m (S1 Table).

### Associations of greenness with breast cancer risk

For each 0.1 unit increase in NDVI, we found a 2.6% increase in BCa risk (Hazard ratio [HR]=1.03, 95%CI 1.00–1.05, Table 2). Women with higher exposure levels had greater risk of breast cancer (HR for 2^^nd^^ vs 1^^st^^ quartile = 1.10, 95% CI 1.01–1.21, HR for 3^^rd^^ vs 1^^st^^ quartile = 1.10, 95% CI 1.00–1.99, HR for 4^^th^^ vs 1^^st^^ quartile = 1.13, 95% CI 1.04–1.24, p for trend = 0.012) (Table 2). Our findings indicated no other associations of any other greenness measures with breast cancer risk.

**Table 2:** Associations of greenness measures with breast cancer risk.

Greenness measure	N with/without breast cancer	HR (95% CI)^a^
Greenspace percentage, buffer 1000m		
Continuous, per 1 unit	5,603/131,132	1.00 (1.00, 1.01)
Q1: ≤ 27.94	1,360/32,823	1.00
Q2: > 27.94- ≤ 42.54	1,433/32,750	1.04 (0.96, 1,12)
Q3: > 42.54– ≤ 60.91	1,385/32,798	0.99 (0.92, 1.07)
Q4: > 60.91	1,425/32,761	1.02 (0.95, 1.10)
P for trend^b^	5,603/131,132	0.987
Greenspace percentage, buffer 300m		
Continuous, per 1 unit	5,603/131,132	1.00 (1.00, 1.01)
Q1: ≤ 17.46	1,364/32,819	1.00
Q2: > 17.46– ≤ 30.14	1,378/32,805	1.01 (0.93, 1.08)
Q3: > 30.14– ≤ 49.24	1,447/32738	1.05 (0.97, 1.13)
Q4: > 49.24	1,414/32,770	1.02 (0.95, 1.10)
P for trend^b^	5,603/131,132	0.473
Natural environment percentage, buffer 1000m		
Continuous, per 1 unit	6,106/148,058	1.00 (1.00, 1.01)
Q1: ≤ 19.98	1,479/37,079	1.00
Q2: > 19.98– ≤ 37.82	1,527/37,009	1.03 (0.96, 1.11)
Q3: > 37.82– ≤ 59.71	1,533/37,009	1.02 (0.95, 1.10)
Q4: > 59.71	1,567/36,961	1.05 (0.98, 1.13)
P for trend^b^	6,106/148,058	0.210
Natural environment percentage, buffer 300m		
Continuous, per 1 unit	6,106/148,058	1.00 (1.00, 1.01)
Q1: ≤ 6.47	1,509/36,936	1.00
Q2: > 6.47– ≤ 19.64	1,457/36,948	0.96 (0.90, 1.03)
Q3: > 19.64– ≤ 40.40	1,557/37,240	1.01 (0.94, 1.09)
Q4: > 40.40	1,583/36,934	1.04 (0.97, 1.12)
P for trend^b^	6,106/148,058	0.088
NDVI mean, buffer 500m		
Continuous, per 0.1 unit	3,944/93,295	1.03 (1.00, 1.05)
Q1: ≤ 0.01	941/23,369	1.00
Q2: > 0.01– ≤ 0.11	1,012/23,297	1.10 (1.01, 1.21)
Q3: > 0.11– ≤ 0.23	979/23,331	1.10 (1.00, 1.20)
Q4: > 0.23	1,012/23,298	1.13 (1.04, 1.24)
P for trend^b^	3,944/93,295	0.012

**Abbreviations**: CI – Confidence interval; HR – Hazard ratio; NDVI – normalized difference vegetation index; Q – quartile.

^a^Adjusted for age, body mass index, race, age at menopause, age at menarche, parity/age at first birth, postmenopausal hormone use, family history of breast cancer, alcohol consumption, and smoking.

^b^P for trend using the median greenness level in each quartile.

### Interactions of greenness with PM_10_ in relation to breast cancer risk

**Fig 2:**
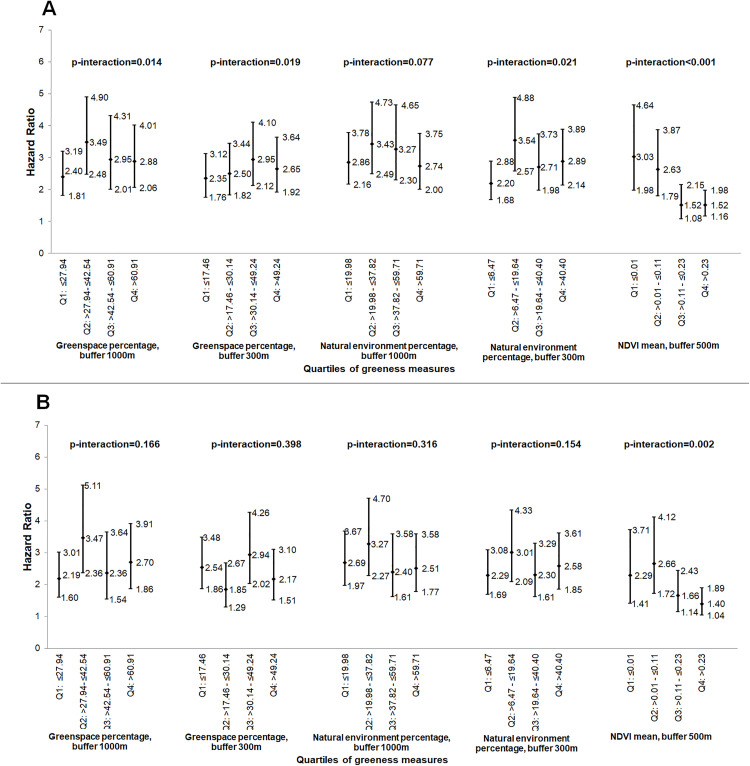
Association of cumulative average PM_10_ (continuous measure, per 10 µg/m^3^) with breast cancer risk, by the quartiles of the greenness measures, (hazard ratios and 95% confidence intervals)^a^. (A) without and (B) with 2-year air pollution exposure lag. NDVI – normalized difference vegetation index; PM_10_ – particulate matter≤10 µm in diameter_;_ Q – quartiles; ^^a^^Risk estimates adjusted for age, body mass index, race, age at menopause, age at menarche, parity/age at first birth, postmenopausal hormone use, family history of breast cancer, alcohol consumption, and smoking; p-interaction: P for interaction between air pollutant measure and greenness measure, with both variables modeled as continuous.

**Fig 3:**
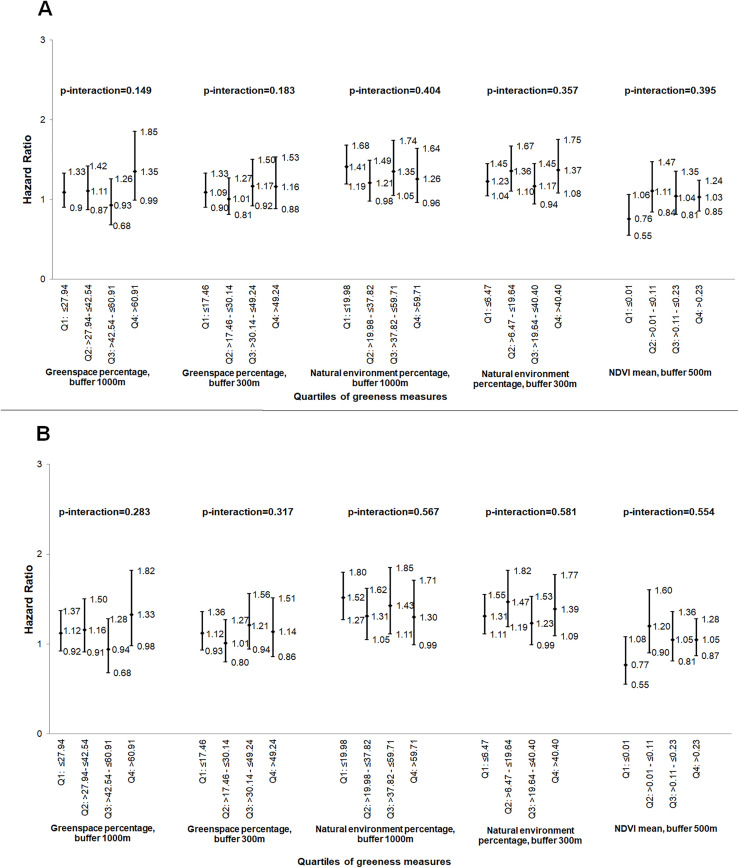
Association of 2007 PM_10_ (continuous measure, per 10 µg/m^3^) with breast cancer risk, by the quartiles of the greenness measures, (hazard ratios and 95% confidence intervals)^a^. (A) without and (B) with 2-year air pollution exposure lag. NDVI – normalized difference vegetation index; PM_10_ – particulate matter≤10 µm in diameter_;_ Q – quartiles; ^^a^^Risk estimates adjusted for age, body mass index, race, age at menopause, age at menarche, parity/age at first birth, postmenopausal hormone use, family history of breast cancer, alcohol consumption, and smoking; p-interaction: P for interaction between air pollutant measure and greenness measure, with both variables modeled as continuous.

**Fig 4:**
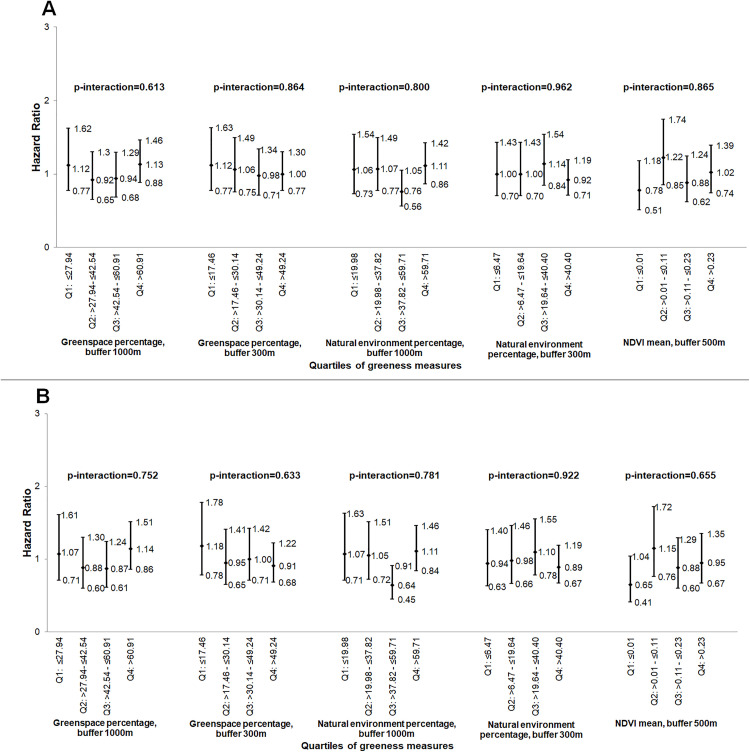
Association of 2010 PM_10_ (continuous measure, per 10 µg/m^3^) with breast cancer risk, by the quartiles of the greenness measures, (hazard ratios and 95% confidence intervals)^a^. (A) without and (B) with 2-year air pollution exposure lag. NDVI – normalized difference vegetation index; PM_10_ – particulate matter≤10 µm in diameter_;_ Q – quartiles; ^^a^^Risk estimates adjusted for age, body mass index, race, age at menopause, age at menarche, parity/age at first birth, postmenopausal hormone use, family history of breast cancer, alcohol consumption, and smoking; p-interaction: P for interaction between air pollutant measure and greenness measure, with both variables modeled as continuous.

As reported previously in this sample [31], PM_10_ was positively associated with breast cancer risk. We found a significant interaction effect between NDVI and cumulative average PM_10_ exposure, modeled as continuous variable, indicating a stronger association of cumulative average PM_10_ with breast cancer risk at lower levels of NDVI (1^^st^^ NDVI quartile: HR per 10 µg/m^^3^^ = 3.03, 95% CI 1.98–4.64) compared to higher levels of NDVI (4^^th^^ NDVI quartile: HR per 10 µg/m^^3^^ = 1.52, 95% CI 1.16–1.98) (p < 0.001, Fig 2). While the interactions between cumulative average PM_10_ and several greenness metrics (greenspace percentage within 1000m, greenspace percentage within 300m, and natural environment percentage within 300m were statistically significant, there was no consistent or dose-dependent trend in the association of cumulative average PM_10_ and breast cancer risk across greenness levels (Fig 2). No other significant interactions between air pollution (on a continuous scale) and greenness measures were found in these analyses (Figs 3 and 4).

When PM_10_ was modeled as quartiles, we found no associations of 2007 PM_10_ or 2010 PM_10_ with breast cancer risk at any of the greenness levels (Tables 3 and 4, respectively). However, women who were in the 4^^th^^ quartile for cumulative average PM_10_ exposure had significantly higher risk of breast cancer as compared to those in the lowest quartile within almost all strata of greenness measures (Table 5). For example, for women in the 1^^st^^, 2^^nd^^, and 3^^rd^^ quartiles of mean NDVI we observed 32%, 37%, and 19% increase in breast cancer risk, for women who were in the 4^^th^^ PM_10_ quartile as compared to those who were in the 1^^st^^ PM_10_ quartile (1^^st^^ NDVI quartile: HR for 4^^th^^ vs. 1^^st^^ PM_10_ quartile = 1.32, 95% CI 1.09–1.59; 2^^nd^^ NDVI quartile: HR = 1.37, 95% CI 1.13–1.66; 3^^rd^^ NDVI quartile: HR = 1.19, 95% CI 1.00–1.67) (Table 5). A marginally significant increase in the risk of breast cancer with increased air pollution exposure was seen among women in the 4^^th^^ quartile of mean NDVI exposure (HR for 4^^th^^ vs 1^^st^^ PM_10_ quartile = 1.13, 95% 0.95–1.36) (Table 5).

**Table 3:** Association of quartiles of 2007 PM_10_ with breast cancer risk, by the quartiles of the greenness measures, without and with 2-year air pollution exposure lag (hazard ratios and 95% confidence intervals)^a^.

Greenness measure	Without air pollution exposure lag				With 2-year air pollution exposure lag			
	2007 PM_10_ quartile (µg/m^3^)			P for trend^c^	2007 PM_10_ quartile (µg/m^3^)			P for trend^c^
	2^nd^ (>20.14- ≤ 21.72)	3^rd^ (>21.72- ≤ 23.54)	4^th^ (>23.54)		2^nd^ (>20.14- ≤ 21.72)	3^rd^ (>21.72- ≤ 23.54)	4^th^ (>23.54)	
Greenspace percentage, buffer 1000m.								
Q1: ≤ 27.94	1.18 (0.80, 1.75)	1.03 (0.71, 1.51)	1.21 (0.84, 1.74)	0.103	1.17 (0.79, 1.73)	1.00 (0.68, 1.45)	1.20 (0.83, 1.72)	0.068
Q2: > 27.94– ≤ 42.54	1.18 (0.92, 1.49)	1.30 (1.03, 1.63)	1.17 (0.93, 1.48)	0.632	1.16 (0.91, 1.48)	1.27 (1.00, 1.61)	1.18 (0.93, 1.50)	0.434
Q3: > 42.54– ≤ 60.91	0.94 (0.81, 1.08)	0.94 (0.81, 1.09)	0.88 (0.72, 1.08)	0.226	0.92 (0.79, 1.06)	0.93 (0.81, 1.08)	0.89 (0.73, 1.09)	0.277
Q4: > 60.91	0.09 (1.08, 1.35)	1.08 (0.93, 1.26)	0.98 (0.61, 1.56)	0.076	1.19 (1.06, 1.33)	1.08 (0.93, 1.26)	0.99 (0.62, 1.58)	0.095
P for interaction ^b^	0.896				0.862			
Greenspace percentage, buffer 300m								
Q1: ≤ 17.46	1.32 (0.97, 1.80)	1.41 (1.05, 1.90)	1.40 (1.05, 1.86)	0.106	1.31 (0.96, 1.8)	1.41 (1.05, 1.90)	1.42 (1.06, 1.90)	0.059
Q2: > 17.46– ≤ 30.14	1.06 (0.87, 1.30)	0.98 (0.80, 1.18)	1.01 (0.84, 1.23)	0.818	1.07 (0.87, 1.3)	0.94 (0.77, 1.14)	1.00 (0.82, 1.22)	0.682
Q3: > 30.14– ≤ 49.24	1.07 (0.92, 1.24)	1.14 (0.98, 1.32)	1.09 (0.92, 1.30)	0.238	1.03 (0.88, 1.2)	1.13 (0.97, 1.32)	1.11 (0.93, 1.32)	0.139
Q4: > 49.24	1.12 (1.00, 1.26)	0.96 (0.83, 1.12)	1.03 (0.79, 1.35)	0.791	1.1 (0.98, 1.24)	0.95 (0.82, 1.11)	1.04 (0.8, 1.37)	0.874
P for interaction ^b^	0.998				0.774			
Natural environment percentage, buffer 1000m								
Q1: ≤ 19.98	1.63 (1.25, 2.11)	1.52 (1.20, 1.95)	1.82 (1.45, 2.28)	<.0001	1.69 (1.29, 2.21)	1.59 (1.24, 2.05)	1.96 (1.55, 2.48)	<.0001
Q2: > 19.98– ≤ 37.82	1.38 (1.16, 1.66)	1.39 (1.17, 1.65)	1.29 (1.08, 1.54)	0.084	1.44 (1.20, 1.73)	1.45 (1.22, 1.73)	1.39 (1.16, 1.67)	0.014
Q3: > 37.82– ≤ 59.71	1.08 (0.95, 1.24)	1.11 (0.97, 1.26)	0.99 (0.82, 1.19)	0.670	1.08 (0.95, 1.24)	1.12 (0.98, 1.28)	1.02 (0.84, 1.23)	0.454
Q4: > 59.71	1.15 (1.03, 1.28)	1.07 (0.98, 1.24)	0.93 (0.63, 1.38)	0.204	1.15 (1.03, 1.28)	1.09 (0.94, 1.26)	0.96 (0.65, 1.42)	0.141
P for interaction ^b^	0.057				0.024			
Natural environment percentage, buffer 300m								
Q1: ≤ 6.47	1.44 (1.15, 1.76)	1.53 (1.25, 1.88)	1.51 (1.24, 1.83)	0.002	1.52 (1.21, 1.91)	1.65 (1.33, 2.04)	1.64 (1.34, 2.01)	0.001
Q2: > 6.47– ≤ 19.64	1.35 (1.14, 1.60)	1.15 (0.97, 1.35)	1.31 (1.11, 1.55)	0.021	1.40 (1.17, 1.66)	1.20 (1.01, 1.42)	1.39 (1.18, 1.65)	0.004
Q3: > 19.64– ≤ 40.40	1.07 (0.94, 1.23)	1.13 (0.99, 1.30)	1.01 (0.86, 1.19)	0.609	1.07 (0.93, 1.23)	1.14 (0.99, 1.31)	1.05 (0.89, 1.24)	0.371
Q4: > 40.40	1.15 (1.03, 1.29)	1.14 (0.99, 1.31)	1.20 (0.95, 1.51)	0.014	1.15 (1.02, 1.29)	1.13 (0.98, 1.30)	1.22 (0.97, 1.54)	0.013
P for interaction ^b^	0.588				0.277			
NDVI mean, buffer 500m								
Q1: ≤ 0.01	0.94 (0.79, 1.13)	0.92 (0.76, 1.10)	0.86 (0.69, 1.06)	0.158	0.92 (0.76, 1.10)	0.91 (0.75, 1.10)	0.86 (0.69, 1.07)	0.186
Q2: > 0.01– ≤ 0.11	0.95 (0.78, 1.17)	0.97 (0.79, 1.17)	1.01 (0.84, 1.22)	0.723	0.98 (0.80, 1.20)	1.01 (0.83, 1.23)	1.07 (0.88, 1.29)	0.347
Q3: > 0.11– ≤ 0.23	1.17 (0.97, 1.41)	0.95 (0.78, 1.15)	1.11 (0.91, 1.34)	0.707	1.16 (0.96, 1.40)	0.95 (0.78, 1.15)	1.11 (0.91, 1.34)	0.681
Q4: > 0.23	1.00 (0.78, 1.29)	1.12 (0.88, 1.42)	1.05 (0.86, 1.29)	0.644	0.98 (0.76, 1.26)	1.05 (0.83, 1.34)	1.04 (0.85, 1.28)	0.580
P for interaction ^b^	0.483				0.691			

**Abbreviations**: NDVI – normalized difference vegetation index; PM_10_ – particulate matter≤10 µm in diameter_;_ Q – quartile.

^a^Risk estimates adjusted for age, body mass index, race, age at menopause, age at menarche, parity/age at first birth, postmenopausal hormone use, family history of breast cancer, alcohol consumption, and smoking; ^b^P for interaction between air pollutant measure and greenness measure, using the respective medians within each of the exposure quartiles; ^c^P for trend using the median air pollutant level within each quartile of greenness measure.

**Table 4:** Association of quartiles of 2010 PM_10_ with breast cancer risk, by the quartiles of the greenness measures, without and with 2-year air pollution exposure lag (hazard ratios and 95% confidence intervals)^a^.

Greenness measure	Without air pollution exposure lag				With 2-year air pollution exposure lag			
	2010 PM_10_ quartile (µg/m^3^)			P for trend^c^	2010 PM_10_ quartile (µg/m^3^)			P for trend^c^
	2^nd^ (>15.22- ≤ 16.01)	3^rd^ (>16.01- ≤ 16.98)	4^th^ (>16.98)		2^nd^ (>15.22- ≤ 16.01)	3^rd^ (>16.01- ≤ 16.98)	4^th^ (>16.98)	
Greenspace percentage, buffer 1000m.								
Q1: ≤ 27.94	0.84 (0.64, 1.11)	0.99 (0.76, 1.28)	0.92 (0.70, 1.20)	0.986	0.87 (0.64, 1.18)	0.99 (0.74, 1.32)	0.93 (0.70, 1.25)	0.999
Q2: > 27.94– ≤ 42.54	0.92 (0.77, 1.11)	0.97 (0.81, 1.16)	0.88 (0.73, 0.06)	0.229	0.92 (0.75, 1.14)	1.02 (0.83, 1.25)	0.89 (0.72, 0.11)	0.375
Q3: > 42.54– ≤ 60.91	0.92 (0.80, 1.07)	0.98 (0.83, 1.14)	0.95 (0.81, 1.11)	0.600	0.89 (0.75, 1.04)	1.02 (0.86, 1.21)	0.87 (0.73, 1.05)	0.263
Q4: > 60.91	1.06 (0.91, 1.22)	0.93 (0.77, 1.13)	1.11 (0.96, 1.29)	0.267	1.05 (0.89, 1.24)	0.85 (0.68, 1.06)	1.15 (0.98, 1.35)	0.234
P for interaction ^b^	0.233				0.410			
Greenspace percentage, buffer 300m								
Q1: ≤ 17.46	0.86 (0.62, 1.19)	1.00 (0.73, 1.37)	0.91 (0.67, 1.26)	0.931	0.82 (0.58, 1.17)	0.98 (0.70, 1.39)	0.92 (0.65, 1.30)	0.690
Q2: > 17.46– ≤ 30.14	1.02 (0.83, 1.25)	1.02 (0.83, 1.25)	0.98 (0.79, 1.20)	0.644	0.93 (0.74, 1.16)	0.96 (0.77, 1.19)	0.89 (0.71, 1.12)	0.366
Q3: > 30.14– ≤ 49.24	0.92 (0.80, 1.06)	0.97 (0.83, 1.13)	0.97 (0.83, 1.13)	0.838	0.94 (0.79, 1.10)	1.04 (0.87, 1.23)	0.98 (0.82, 1.16)	0.968
Q4: > 49.24	1.00 (0.85, 1.18)	0.99 (0.82, 1.21)	1.03 (0.88, 1.20)	0.768	1.01 (0.85, 1.21)	0.94 (0.75, 1.17)	0.99 (0.83, 1.17)	0.803
P for interaction ^b^	0.418				0.960			
Natural environment percentage, buffer 1000m								
Q1: ≤ 19.98	0.78 (0.59, 1.03)	0.89 (0.68, 1.16)	0.83 (0.64, 1.08)	0.546	0.77 (0.56, 1.05)	0.88 (0.66, 1.18)	0.85 (0.63, 1.14)	0.923
Q2: > 19.98– ≤ 37.82	1.00 (0.83, 1.21)	1.10 (0.91, 1.33)	1.00 (0.82, 1.21)	0.944	1.05 (0.85, 1.31)	1.19 (0.96, 1.48)	1.04 (0.83, 1.30)	0.858
Q3: > 37.82– ≤ 59.71	0.89 (0.77, 1.03)	0.94 (0.81, 1.09)	0.86 (0.74, 1.01)	0.097	0.84 (0.72, 0.99)	0.95 (0.80, 1.12)	0.77 (0.64, 0.92)	0.012
Q4: > 59.71	1.04 (0.90, 1.20)	0.92 (0.76, 1.11)	1.10 (0.95, 1.28)	0.311	1.04 (0.88, 1.22)	0.87 (0.70, 1.07)	1.14 (0.97, 1.33)	0.270
P for interaction ^b^	0.293				0.562			
Natural environment percentage, buffer 300m								
Q1: ≤ 6.47	0.72 (0.54, 0.96)	0.83 (0.63, 1.1)	0.75 (0.56, 0.99)	0.245	0.66 (0.48, 0.90)	0.75 (0.56, 1.01)	0.70 (0.52, 0.94)	0.373
Q2: > 6.47– ≤ 19.64	0.94 (0.76, 1.16)	0.98 (0.8, 1.21)	0.94 (0.75, 1.16)	0.655	0.87 (0.69, 1.10)	0.99 (0.79, 1.25)	0.90 (0.71, 1.14)	0.692
Q3: > 19.64– ≤ 40.40	1.01 (0.87, 1.16)	1.01 (0.87, 1.18)	1.01 (0.87, 1.19)	0.866	1.03 (0.87, 1.21)	1.05 (0.88, 1.24)	1.02 (0.85, 1.22)	0.815
Q4: > 40.40	0.97 (0.83, 1.13)	1.01 (0.83, 1.22)	1.02 (0.87, 1.18)	0.884	0.99 (0.84, 1.18)	1.03 (0.83, 1.27)	0.98 (0.83, 1.16)	0.865
P for interaction ^b^	0.454				0.844			
NDVI mean, buffer 500m								
Q1: ≤ 0.01	0.86 (0.71, 1.04)	0.91 (0.75, 1.09)	0.80 (0.65, 0.99)	0.062	0.78 (0.63, 0.97)	0.89 (0.73, 1.10)	0.76 (0.60, 0.96)	0.045
Q2: > 0.01– ≤ 0.11	0.96 (0.97, 1.15)	1.20 (1.00, 1.43)	1.08 (0.89, 1.32)	0.184	1.00 (0.81, 1.23)	1.19 (0.97, 1.46)	1.08 (0.86, 1.34)	0.313
Q3: > 0.11– ≤ 0.23	1.01 (0.84, 1.21)	0.99 (0.83, 1.18)	0.99 (0.82, 1.18)	0.856	1.02 (0.84, 1.25)	0.99 (0.81, 1.21)	1.01 (0.83, 1.24)	0.974
Q4: > 0.23	1.06 (0.86, 1.31)	1.05 (0.86, 1.29)	1.01 (0.85, 1.21)	0.979	1.05 (0.83, 1.33)	1.02 (0.82, 1.26)	0.97 (0.8, 1.18)	0.666
P for interaction ^b^	0.553				0.539			

**Abbreviations**: NDVI – normalized difference vegetation index; PM_10_ – particulate matter≤10 µm in diameter_;_ Q – quartiles.

^a^Risk estimates adjusted for age, body mass index, race, age at menopause, age at menarche, parity/age at first birth, postmenopausal hormone use, family history of breast cancer, alcohol consumption, and smoking; ^b^P for interaction between air pollutant measure and greenness measure, using the respective medians within each of the exposure quartiles; ^c^P for trend using the median air pollutant level within each quartile of greenness measure.

**Table 5:** Association of quartiles of cumulative average PM_10_ with breast cancer risk, by the quartiles of the greenness measures, without and with 2-year air pollution exposure lag (hazard ratios and 95% confidence intervals)^a^.

Greenness measure	Without air pollution exposure lag				With 2-year air pollution exposure lag			
	Cumulative average PM_10_ quartile (µg/m^3^)			P for trend	Cumulative average PM_10_ quartile (µg/m^3^)			P for trend
	2^nd^ (>17.92- ≤ 19.04)	3^rd^ (>19.04- ≤ 20.25)	4^th^ (>20.25)		2^nd^ (>17.92- ≤ 19.04)	3^rd^ (>19.04- ≤ 20.25)	4^th^ (>20.25)	
Greenspace percentage, buffer 1000m.								
Q1: ≤ 27.94	0.97 (0.66, 1.42)	0.98 (0.68, 1.41)	1.22 (0.85, 1.75)	<0.001	0.92 (0.61, 1.37)	0.88 (0.60, 1.30)	1.08 (0.74, 1.58)	0.015
Q2: > 27.94– ≤ 42.54	1.19 (0.95, 1.49)	1.12 (0.90, 1.40)	1.48 (1.19, 1.84)	<0.001	1.22 (0.94, 1.58)	1.20 (0.93, 1.54)	1.51 (1.17, 1.94)	0.001
Q3: > 42.54– ≤ 60.91	0.97 (0.84, 1.11)	1.08 (0.92, 1.25)	1.45 (1.22, 1.71)	<0.001	0.94 (0.80, 1.10)	1.07 (0.9, 1.26)	1.32 (1.09, 1.59)	0.003
Q4: > 60.91	1.15 (1.01, 1.31)	1.25 (1.06, 1.47)	1.94 (1.61, 2.33)	<0.001	1.13 (0.98, 1.30)	1.26 (1.05, 1.51)	1.92 (1.56, 2.35)	<0.001
P for interaction	0.047				0.101			
Greenspace percentage, buffer 300m								
Q1: ≤ 17.46	1.23 (0.88, 1.72)	1.35 (0.98, 1.86)	1.56 (1.14, 2.14)	<0.001	1.09 (0.77, 1.56)	1.22 (0.87, 1.72)	1.41 (1.01, 1.97)	<0.001
Q2: > 17.46– ≤ 30.14	1.11 (0.90, 1.36)	0.96 (0.78, 1.17)	1.33 (1.08, 1.63)	<0.001	1.07 (0.85, 1.34)	0.93 (0.74, 1.17)	1.21 (0.97, 1.51)	0.037
Q3: > 30.14– ≤ 49.24	1.03 (0.89, 1.20)	1.17 (.001, 1.36)	1.48 (1.26, 1.73)	<0.001	1.03 (0.87, 1.22)	1.25 (1.05, 1.48)	1.45 (1.21, 1.74)	<0.001
Q4: > 49.24	1.09 (0.95, 1.24)	1.14 (0.97, 1.35)	1.83 (1.54, 2.18)	<0.001	1.08 (0.94, 1.25)	1.09 (0.90, 1.31)	1.67 (1.37, 2.04)	<0.001
P for interaction	0.056				0.359			
Natural environment percentage, buffer 1000m								
Q1: ≤ 19.98	0.78 (0.56, 1.10)	0.75 (0.54, 1.03)	1.03 (0.75, 1.40)	<0.001	0.71 (0.49, 1.01)	0.70 (0.50, 0.99)	0.92 (0.66, 1.28)	<0.001
Q2: > 19.98– ≤ 37.82	1.26 (1.02, 1.55)	1.30 (1.07, 1.60)	1.69 (1.38, 2.07)	<0.001	1.29 (1.02, 1.63)	1.36 (1.08, 1.71)	1.70 (1.35, 2.15)	<0.001
Q3: > 37.82– ≤ 59.71	1.07 (0.94, 1.22)	1.14 (0.99, 1.32)	1.49 (1.27, 1.74)	<0.001	1.04 (0.90, 1.21)	1.11 (0.95, 1.30)	1.28 (1.07, 1.53)	<0.001
Q4: > 59.71	1.11 (0.98, 1.25)	1.23 (1.05, 1.44)	1.94 (1.63, 2.31)	<0.001	1.10 (0.96, 1.26)	1.21 (1.02, 1.45)	1.94 (1.60, 2.36)	<0.001
P for interaction	0.162				0.227			
Natural environment percentage, buffer 300m								
Q1: ≤ 6.47	1.33 (0.99, 1.78)	1.31 (0.99, 1.74)	1.55 (1.18, 2.05)	0.001	1.11 (0.81, 1.51)	1.15 (0.85, 1.54)	1.35 (1.01, 1.81)	0.001
Q2: > 6.47– ≤ 19.64	1.15 (0.96, 1.39)	1.04 (0.86, 1.26)	1.50 (1.25, 1.81)	<0.001	1.17 (0.95, 1.45)	1.09 (0.88, 1.34)	1.43 (1.16, 1.76)	<0.001
Q3: > 19.64– ≤ 40.40	1.00 (0.87, 1.15)	1.12 (0.97, 1.30)	1.44 (1.24, 1.67)	<0.001	1.01 (0.87, 1.18)	1.15 (0.98, 1.35)	1.36 (1.15, 1.61)	0.001
Q4: > 40.40	1.11 (0.98, 1.25)	1.23 (1.06, 1.43)	1.92 (1.62, 2.26)	<0.001	1.10 (0.96, 1.26)	1.20 (1.01, 1.42)	1.85 (1.53, 2.22)	<0.001
P for interaction	0.081				0.225			
NDVI mean, buffer 500m								
Q1: ≤ 0.01	0.87 (0.72, 1.04)	1.00 (0.83, 1.20)	1.32 (1.09, 1.59)	<0.001	0.80 (0.65, 0.98)	0.96 (0.79, 1.18)	1.14 (0.93, 1.41)	0.072
Q2: > 0.01– ≤ 0.11	1.18 (0.97, 1.45)	1.24 (1.02, 1.50)	1.37 (1.13, 1.66)	0.001	1.26 (1.01, 1.59)	1.31 (1.05, 1.63)	1.40 (1.12, 1.74)	0.005
Q3: > 0.11– ≤ 0.23	1.00 (0.84, 1.20)	0.95 (0.79, 1.14)	1.19 (1.00, 1.42)	0.061	0.96 (0.79, 1.18)	0.96 (0.78, 1.17)	1.22 (1.00, 1.48)	0.037
Q4: > 0.23	0.94 (0.74, 1.20)	0.94 (0.75, 1.18)	1.13 (0.95, 1.36)	0.050	0.95 (0.72, 1.24)	0.90 (0.71, 1.16)	1.06 (0.87, 1.29)	0.330
P for interaction	0.061				0.195			

**Abbreviations**: NDVI – normalized difference vegetation index; PM_10_ – particulate matter≤10 µm in diameter_;_ Q – quartiles.

^a^Risk estimates adjusted for age, body mass index, race, age at menopause, age at menarche, parity/age at first birth, postmenopausal hormone use, family history of breast cancer, alcohol consumption, and smoking; ^b^ P for interaction between air pollutant measure and greenness measure, using the respective medians within each of the exposure quartiles; ^c^P for trend using the median air pollutant level within each quartile of greenness measure.

### Secondary analyses

Similar to findings from the main analysis, in the lagged analysis we found that the association of cumulative average PM_10_ with breast cancer risk remained stronger at lower NDVI levels as compared to higher NDVI levels (p = 0.002) (Fig 2), and that the cumulative average PM_10_ was positively associated with breast cancer risk within most of the greenness strata (Table 5).

The association between NDVI and invasive breast cancer became stronger as compared to the main analysis (HR per 0.1-unit NDVI change = 1.04, 95% CI 1.02–1.06) (S2 Table). There were no other associations found between greenness measures and invasive breast cancer risk.

Lower levels of NDVI had stronger association of cumulative average PM_10_ with invasive breast cancer risk (for 1^^st^^ NDVI quartile: HR per 10 µg/m^^3^^ = 3.10, 95% CI 1.94–4.95) compared to higher levels of NDVI (for 4^^th^^ NDVI quartile: HR per 10 µg/m^^3^^ = 1.66, 95% CI 1.24–2.21) (S3 Table). Similar to the overall analyses, there was not a clear trend for the association of cumulative average PM_10_ with invasive breast cancer risk for the other greenness measures. No other interactions were significant.

When PM_10_ was modeled as quartiles (S4, S5 and S6 Tables), women who were in the 4^^th^^ quartile for cumulative average PM_10_ exposure remained at higher risk of invasive breast cancer as compared to those who were in the 1^^st^^ PM_10_ quartile within almost all strata of greenness measures (S6 Table).

In the lagged analysis, the association between cumulative average PM_10_ and invasive breast cancer remained stronger for lower levels of NDVI (1^^st^^ NDVI quartile: HR per 10 µg/m^^3^^ = 2.17, 95% CI 1.27–3.70) compared to higher NDVI levels (4^^th^^ NDVI quartile: HR per 10 µg/m^^3^^ = 1.58, 95% CI 1.14–2.18). There were no other interactions between any other PM_10_ and greenness measures (S3 Table). When PM_10_ was modeled as quartiles, cumulative average PM_10_ was positively associated with invasive breast cancer risk in most of the greenness strata (S6 Table). A trend of higher breast cancer risk for women in the 4^^th^^ vs 1^^st^^ quartile for cumulative average PM_10_ exposure was observed within each of the greenness levels; however, not all were statistically significant (S6 Table).

## Discussion

Our findings indicated that NDVI was positively associated with postmenopausal breast cancer risk. The association between cumulative average PM₁₀ and breast cancer risk was stronger among individuals residing in areas with lower NDVI levels compared to those in areas with higher NDVI. Greenness percentage and natural environment percentage were not associated with breast cancer risk.

Positive associations of NDVI with breast cancer were found in some studies [24], but not all studies [20,22,23] that investigated the association of greenness with breast cancer risk. These inconsistencies between reported associations between greenness and breast cancer risk, including from our study, may also be due to the differences in study designs, geographic context, exposure assessment methods, and participants characteristics. Consistent with our findings, a study conducted by O’Callaghan-Gordo et al. [24] found that on average NDVI within the 300m-500m buffer was associated with increased risk of breast cancer (Odds ratio [OR] per 1-Interquartile range [IQR]=1.17, 95% CI 1.02–1.34) [24]. In a study including 6,500 women with breast cancer [22], the authors found that the risk of breast cancer was reduced by 4% for a 1-IQR increase of NDVI (HR per 1-IQR = 0.96, 95% CI 0.92-0.99); however, they did not account for risk factors for breast cancer nor examined the associations separately by menopausal status. Hart et al. [23] found that women living in the 5^^th^^ quintile of cumulative average NDVI had lower risk of breast cancer compared to those in the 1^^st^^ quintile, adjusting for a wide range of breast cancer risk factors (HR = 0.87, 95% CI 0.75–1.01, p for trend = 0.02). However, the cohort was limited to specific sub-population of women (female nurses), which may limit the generalizability to more diverse populations. In addition, Villeneuve et al. [22] and James et al. [23] used prospective cohort designs in large populations in Canada and the United States, respectively, assessing long-term residential greenness using satellite-derived NDVI. In contrast, O’Callaghan et al. [20] and O’Callaghan-Gordo et al. [24] conducted case-control studies within Spanish populations, with the former assessing proximity to green spaces rather than NDVI.

The use of different units for the greenness measures (e.g., percent of greenspace and natural environment vs. NDVI measured using an index measure), which might explain why NDVI might be associated with breast cancer risk in contrast to greenspace percentage and natural environment percentage. In addition, the choice of buffer distance might represent different exposure pathways, which a shorter distance capturing nearby and frequent contacts with greenness [33]. Finally, NDVI focuses on the health and quantity of vegetation while the other greenness measures included in these analyses are reflective of one area covered by green space.

To the best of our knowledge, no other study looked at associations of PM_10_ with postmenopausal breast cancer by levels of NDVI. We found that the association of cumulative average PM_10_ with breast cancer risk was stronger at lower levels of NDVI, suggesting the potential modifying effect of greenness on the association of PM_10_ and breast cancer risk. Possible mechanisms through which greenness might modify this association include reduction in the level of pollutants, especially PM, and increase in pollutants disperse [41–43].

Among postmenopausal women, our study is the largest to date to examine the interaction between greenness and air pollution in relation to breast cancer risk. UK Biobank is a large population-based prospective cohort with rigorous protocols, regular updates of health outcomes, and valid methods for estimating the exposure measures, such as air pollution and greenness. Several limitations need to be considered. Only data from the baseline assessment for greenspace percentage, natural environment percentage, and covariates were used in analyses; however, repeated measures from baseline and follow-up assessments were highly correlated. Greenspace percentage was not available for participants outside England, and NDVI was not available for northern England and Scotland, which might affect the generalizability of the observed associations. As the participant’s residential address at the time of enrollment in the UK Biobank was used to calculate the concentrations of air, we could have been potentially misclassifying the participants based on their exposure due to other activities away from their residential address. However, other studies suggest that similar effects were observed when analyses included both time at home and work as compared to analyses including only time at home [44,45]. Next, despite the difference in the average follow-up time between breast cancer cases (5.4 years) and women without breast cancer (10.7 years), the proportion of women with available data on each of the air pollution measures was similar between the two groups (2007 PM_10_: 99.5% for both cases and controls; 2010 PM_10_: 92.5% for cases and 94.6% for controls; cumulative average PM_10_: 99.7% for both cases and controls). Additionally, 92.5% of the controls and 94.7% of the cases had the cumulative average PM_10_ estimate based on both 2007 PM_10_ and 2010 PM_10_ measures. Finally, we could not differentiate among breast cancer subtypes because the data was not available in the UK Biobank. As breast cancer subtypes may have distinct etiologies and environmental risk factors, future studies with access to richer clinical data are needed to explore subtype-specific associations with air pollution and greenness exposure.

## Conclusion

In this study, we found a positive association between NDVI, a common measure of greenness, and postmenopausal breast cancer risk. Additionally, we observed a significant interaction between cumulative average PM₁₀ and NDVI, where the association between PM₁₀ exposure and breast cancer risk was stronger among women living in areas with lower greenness compared to those in greener environments. These findings suggest that greenness may not have a uniformly protective effect and that its role in modifying the health impacts of air pollution may be more complex than previously thought.

Importantly, our results contribute to the very limited body of evidence examining how environmental exposures such as greenness and air pollution interact in relation to breast cancer risk, particularly among postmenopausal women. The differential associations observed across various greenness metrics also underscore the need for careful selection and interpretation of environmental exposure indicators in future research.

Further studies are warranted to replicate these findings in diverse populations and geographic settings, and to explore underlying biological mechanisms. A better understanding of how greenness and air pollution jointly influence breast cancer risk could inform public health strategies aimed at reducing environmental risk factors and promoting healthier living environments for women.

## Supporting information

S1 Table Distribution of greenness and PM_10_ measures in the study sample. (PDF)

S2 Table Associations of greenness measures with invasive breast cancer risk. (PDF)

S3 Table Association of PM_10_ (continuous measure, per 10 µg/m^3^) with invasive breast cancer risk, by the quartiles of the greenness measures, without and with 2-year air pollution exposure lag (hazard ratios and 95% confidence intervals). (PDF)

S4 Table Association of quartiles of 2007 PM_10_ with invasive breast cancer risk, by the quartiles of the greenness measures, without and with 2-year air pollution exposure lag (hazard ratios and 95% confidence intervals). (PDF)

S5 Table Association of quartiles of 2010 PM_10_ with invasive breast cancer risk, by the quartiles of the greenness measures, without and with 2-year air pollution exposure lag (hazard ratios and 95% confidence intervals). (PDF)

S6 Table Association of quartiles of cumulative average PM_10_ with breast cancer risk, by the quartiles of the greenness measures, without and with 2-year air pollution exposure lag (hazard ratios and 95% confidence intervals). (PDF)
