# Supplementary material for: Greenness and its interaction with air pollution in relation to postmenopausal breast cancer risk in UK Biobank
Source: PLoS One. 2025 Nov 12;20(11):e0334744. doi: 10.1371/journal.pone.0334744 (PMC12611134; doi:10.1371/journal.pone.0334744)
Supplement: S1 Table — (PDF) [file pone.0334744.s001.pdf]

S1 Table. Distribution of greenness and PM_{10} measures in the study sample

Greenness measure	N	Mean	SD	Minimum	25th Percentile	Median	75th Percentile	Maximum
Greenspace percentage, buffer 1000m	136,735	45.68	21.72	4.96	27.94	42.54	60.91	99.18
Greenspace percentage, buffer 300m	136,735	35.77	23.41	0.23	17.46	30.14	49.24	99.18
Natural environment percentage, buffer 1000m	154,164	41.46	25.61	0.00	19.98	37.82	59.71	100.00
Natural environment percentage, buffer 300m	154,164	26.72	25.25	0.00	6.47	19.64	40.40	100.00
NDVI mean, buffer 500m	97,239	0.11	0.14	-0.49	0.01	0.11	0.23	0.54
Particulate matter (PM_{10}); 2007	153,983	22.00	2.86	11.81	20.14	21.72	23.54	36.56
Particulate matter (PM_{10}); 2010	143,305	16.20	1.89	11.78	15.22	16.01	16.98	30.65

Abbreviations: m - meters; N – number of participants; NDVI - normalized difference vegetation index; PM_{10} - particulate matter≤10 μm in diameter; SD - standard deviation
