# Supplementary material for: Greenness and its interaction with air pollution in relation to postmenopausal breast cancer risk in UK Biobank
Source: PLoS One. 2025 Nov 12;20(11):e0334744. doi: 10.1371/journal.pone.0334744 (PMC12611134; doi:10.1371/journal.pone.0334744)
Supplement: S2 Table — (PDF) [file pone.0334744.s002.pdf]

S2 Table. Associations of greenness measures with invasive breast cancer risk

Greenness measure	N with/without breast cancer	HR (95% CI)
Greenspace percentage, buffer 1000m		
Continuous, per 1 unit	4,731/131,132	1.00 (0.99, 1.01)
Q1: ≤27.94	1,138/32,823	1.00
Q2: >27.94 - ≤42.54	1,217/32,750	1.05 (0.97, 1.10)
Q3: >42.54 - ≤60.91	1,184/32,798	1.01 (0.93, 1.10)
Q4: >60.91	1,192/32,761	1.02 (0.94, 1.11)
P for trend[b]	4,731/131,132	0.964
Greenspace percentage, buffer 300m		
Continuous, per 1 unit	4,731/131,132	1.003 (1.00, 1.01)
Q1: ≤17.46	1,144/32,819	1.00
Q2: >17.46 - ≤30.14	1,157/32,805	1.00 (0.93, 1.09)
Q3: >30.14 - ≤49.24	1,235/32,738	1.07 (0.98, 1.15)
Q4: >49.24	1,195/32,770	1.03 (0.95, 1.12)
P for trend[b]	4,731/131,132	0.388
Natural environment percentage, buffer 1000m		
Continuous, per 1 unit	5,162/148,058	1.00 (1.00, 1109)
Q1: ≤19.98	1,245/37,079	1.00
Q2: >19.98 - ≤37.82	1,300/37,009	1.04 (0.96, 1.12)
Q3: >37.82 - ≤59.71	1,295/37,009	1.02 (0.95, 1.11)
Q4: >59.71	1,322/36,961	1.05 (0.97, 1.14)
P for trend[b]	5,162/148,058	0.280
Natural environment percentage, buffer 300m		
Continuous, per 1 unit	5,162/148,058	1.01 (1.00, 1.01)
Q1: ≤6.47	1,273/36,936	1.00
Q2: >6.47 - ≤19.64	1,227/36,948	0.96 (0.89, 1.04)
Q3: >19.64 - ≤40.40	1,318/37,240	1.01 (0.94, 1.10)
Q4: >40.40	1,344/36,934	1.05 (0.97, 1.13)
P for trend[b]	5,162/148,058	0.080
NDVI mean, buffer 500m		
Continuous, per 0.1 unit	3,305/93,295	1.04 (1.02, 1.06)
Q1: ≤0.01	768/23,369	1.00
Q2: >0.01 - ≤0.11	848/23,297	1.13 (1.03, 1.25)
Q3: >0.11 - ≤0.23	827/23,331	1.14 (1.03, 1.25)
Q4: >0.23	862/23,298	1.19 (1.08, 1.31)
P for trend[b]	3,305/93,295	0.001

Abbreviations: CI - Confidence interval; HR - Hazard ratio; NDVI - normalized difference vegetation index; Q - quartile

[a]Adjusted for age, body mass index, race, age at menopause, age at menarche, parity/age at first birth, postmenopausal hormone use, family history of breast cancer, alcohol consumption, and smoking; [b]P for trend using the median greenness level in each quartile
