# Supplementary material for: Greenness and its interaction with air pollution in relation to postmenopausal breast cancer risk in UK Biobank
Source: PLoS One. 2025 Nov 12;20(11):e0334744. doi: 10.1371/journal.pone.0334744 (PMC12611134; doi:10.1371/journal.pone.0334744)
Supplement: S3 Table — (PDF) [file pone.0334744.s003.pdf]

**S3 Table. Association of $PM_{10}$ (continuous measure, per 10 µg/$m^3$) with invasive breast cancer risk, by the quartiles of the greenness measures, without and with 2-year air pollution exposure lag (hazard ratios and 95% confidence intervals)[a]**

| Greenness measure | Without air pollution exposure lag | | | With 2-year air pollution exposure lag | | |
|---|---|---|---|---|---|---|
| | 2007 $PM_{10}$ | 2010 $PM_{10}$ | Cumulative average $PM_{10}$ | 2007 $PM_{10}$ | 2010 $PM_{10}$ | Cumulative average $PM_{10}$ |
| Greenspace percentage, buffer 1000m | | | | | | |
| Q1: ≤27.94 | 1.18 (0.95, 1.46) | 1.24 (0.83, 1.85) | 2.65 (1.95, 3.62) | 1.21 (0.98, 1.51) | 1.23 (0.79, 1.91) | 2.5 (1.78, 3.53) |
| Q2: >27.94- ≤42.54 | 1.09 (0.83, 1.43) | 0.89 (0.61, 1.3) | 3.62 (2.50, 5.23) | 1.14 (0.87, 1.50) | 0.86 (0.56, 1.31) | 3.42 (2.25, 5.20) |
| Q3: >42.54 - ≤60.91 | 0.96 (0.68, 1.34) | 0.90 (0.63, 1.27) | 3.12 (2.07, 4.70) | 0.96 (0.68, 1.34) | 0.82 (0.55, 1.21) | 2.55 (1.60, 4.06) |
| Q4: >60.91 | 1.28 (0.91, 1.79) | 1.15 (0.87, 1.51) | 2.81 (1.96, 4.05) | 1.24 (0.88, 1.75) | 1.15 (0.84, 1.56) | 2.53 (1.68, 3.81) |
| P for interaction [b] | 0.291 | 0.755 | 0.031 | 0.530 | 0.906 | 0.387 |
| Greenspace percentage, buffer 300m | | | | | | |
| Q1: ≤17.46 | 1.12 (0.91, 1.38) | 1.10 (0.73, 1.66) | 2.39 (1.75, 3.26) | 1.16 (0.94, 1.43) | 1.20 (0.77, 1.87) | 2.61 (1.86, 3.66) |
| Q2: >17.46 - ≤30.14 | 1.11 (0.87, 1.42) | 1.22 (0.84, 1.77) | 3.11 (2.20, 4.39) | 1.11 (0.86, 1.42) | 1.11 (0.73, 1.68) | 2.26 (1.53, 3.35) |
| Q3: >30.14 - ≤49.24 | 1.20 (0.92, 1.57) | 0.93 (0.66, 1.31) | 2.95 (2.06, 4.22) | 1.24 (0.94, 1.62) | 0.92 (0.63, 1.35) | 2.87 (1.91, 4.29) |
| Q4: >49.24 | 1.11 (0.82, 1.51) | 1.01 (0.76, 1.35) | 2.67 (1.89, 3.77) | 1.09 (0.80, 1.48) | 0.90 (0.66, 1.25) | 2.12 (1.43, 3.15) |
| P for interaction [b] | 0.228 | 0.786 | 0.021 | 0.404 | 0.548 | 0.530 |
| Natural environment percentage, buffer 1000m | | | | | | |
| Q1: ≤19.98 | 1.44 (1.20, 1.74) | 1.24 (0.83, 1.85) | 3.09 (2.28, 4.19) | 1.55 (1.28, 1.87) | 1.33 (0.85, 2.07) | 3.02 (2.16, 4.24) |
| Q2: >19.98 - ≤37.82 | 1.22 (0.97, 1.53) | 1.01 (0.70, 1.44) | 3.60 (2.55, 5.10) | 1.33 (1.05, 1.68) | 0.95 (0.63, 1.42) | 3.25 (2.19, 4.82) |
| Q3: >37.82 - ≤59.71 | 1.38 (1.05, 1.81) | 0.73 (0.51, 1.03) | 3.29 (2.24, 4.82) | 1.45 (1.10, 1.92) | 0.62 (0.42, 0.91) | 2.46 (1.60, 3.79) |
| Q4: >59.71 | 1.23 (0.92, 1.65) | 1.13 (0.87, 1.49) | 2.81 (1.99, 3.95) | 1.27 (0.94, 1.70) | 1.14 (0.84, 1.54) | 2.52 (1.72, 3.71) |
| P for interaction [b] | 0.555 | 0.939 | 0.136 | 0.794 | 0.941 | 0.572 |
| Natural environment percentage, buffer 300m | | | | | | |
| Q1: ≤6.47 | 1.27 (1.06, 1.52) | 1.07 (0.72, 1.57) | 2.4 (1.79, 3.21) | 1.37 (1.14, 1.64) | 1.04 (0.68, 1.60) | 2.51 (1.82, 3.47) |
| Q2: >6.47 - ≤19.64 | 1.40 (1.12, 1.75) | 1.11 (0.76, 1.62) | 4.04 (2.85, 5.72) | 1.51 (1.20, 1.90) | 1.16 (0.76, 1.77) | 3.60 (2.43, 5.33) |
| Q3: >19.64 - ≤40.40 | 1.14 (0.90, 1.44) | 0.99 (0.71, 1.39) | 2.56 (1.81, 3.61) | 1.21 (0.95, 1.54) | 0.89 (0.61, 1.31) | 1.99 (1.35, 2.96) |
| Q4: >40.40 | 1.38 (1.06, 1.79) | 1.00 (0.76, 1.33) | 2.99 (2.16, 4.13) | 1.38 (1.06, 1.80) | 0.95 (0.69, 1.29) | 2.61 (1.82, 3.75) |
| P for interaction [b] | 0.486 | 0.844 | 0.042 | 0.788 | 0.861 | 0.347 |
| NDVI mean, buffer 500m | | | | | | |
| Q1: ≤0.01 | 0.80 (0.56, 1.15) | 0.70 (0.44, 1.11) | 3.10 (1.94, 4.95) | 0.80 (0.55, 1.16) | 0.58 (0.34, 0.97) | 2.17 (1.27, 3.70) |
| Q2: >0.01 - ≤0.11 | 1.15 (0.84, 1.56) | 1.30 (0.88, 1.93) | 2.78 (1.82, 4.25) | 1.23 (0.90, 1.68) | 1.22 (0.78, 1.89) | 2.81 (1.74, 4.53) |
| Q3: >0.11 - ≤0.23 | 0.96 (0.72, 1.28) | 0.83 (0.57, 1.21) | 1.45 (0.99, 2.11) | 0.97 (0.73, 1.29) | 0.81 (0.53, 1.23) | 1.60 (1.06, 2.42) |
| Q4: >0.23 | 1.08 (0.88, 1.32) | 1.12 (0.80, 1.58) | 1.66 (1.24, 2.21) | 1.10 (0.90, 1.36) | 1.06 (0.73, 1.56) | 1.58 (1.14, 2.18) |
| P for interaction [b] | 0.352 | 0.419 | 0.001 | 0.451 | 0.274 | 0.024 |

**Abbreviations**: NDVI - normalized difference vegetation index; $PM_{10}$ - particulate matter≤10 µm in diameter; Q - quartiles

[a]Risk estimates adjusted for age, body mass index, race, age at menopause, age at menarche, parity/age at first birth, postmenopausal hormone use, family history of breast cancer, alcohol consumption, and smoking;

[b]P for interaction between air pollutant measure and greenness measure, with both variables modeled as continuous
