# Supplementary material for: Greenness and its interaction with air pollution in relation to postmenopausal breast cancer risk in UK Biobank
Source: PLoS One. 2025 Nov 12;20(11):e0334744. doi: 10.1371/journal.pone.0334744 (PMC12611134; doi:10.1371/journal.pone.0334744)
Supplement: S4 Table — (PDF) [file pone.0334744.s004.pdf]

**S4 Table. Association of quartiles of 2007 $PM_{10}$ with invasive breast cancer risk, by the quartiles of the greenness measures, without and with 2-year air pollution exposure lag (hazard ratios and 95% confidence intervals)[a]**

| Greenness measure | Without air pollution exposure lag | | | | With 2-year air pollution exposure lag | | | |
|---|---|---|---|---|---|---|---|---|
| | 2007 $PM_{10}$ quartile (μg/m$^3$) | | | | 2007 $PM_{10}$ quartile (μg/m$^3$) | | | |
| | 2nd (>20.14-≤21.72) | 3rd (>21.72-≤23.54) | 4th ( >23.54) | P for trend[c] | 2nd (>20.14-≤21.72) | 3rd (>21.72-≤23.54) | 4th ( >23.54) | P for trend[c] |
| Greenspace percentage, buffer 1000m. | | | | | | | | |
| Q1: ≤27.94 | 1.13 (0.74, 1.72) | 0.98 (0.65, 1.47) | 1.17 (0.79, 1.74) | 0.079 | 1.11 (0.72, 1.69) | 0.95 (0.63, 1.42) | 1.16 (0.78, 1.72) | 0.057 |
| Q2: >27.94 - ≤42.54 | 1.20 (0.93, 1.57) | 1.32 (1.03, 1.71) | 1.19 (0.92, 1.54) | 0.629 | 1.71 (0.90, 1.53) | 1.28 (0.99, 1.65) | 1.19 (0.92, 1.54) | 0.468 |
| Q3: >42.54 - ≤60.91 | 0.94 (0.80, 1.09) | 0.99 (0.85, 1.16) | 0.88 (0.70, 1.09) | 0.410 | 0.91 (0.78, 1.07) | 0.98 (0.84, 1.14) | 0.88 (0.71, 1.10) | 0.459 |
| Q4: >60.91 | 1.19 (1.05, 1.35) | 1.07 (0.91, 1.26) | 1.04 (0.63, 1.71) | 0.119 | 1.16 (1.02, 1.31) | 1.07 (0.90, 1.26) | 1.05 (0.64, 1.73) | 0.160 |
| P for interaction[b] | 0.767 | | | | 0.969 | | | |
| Greenspace percentage, buffer 300m | | | | | | | | |
| Q1: ≤17.46 | 1.30 (0.93, 1.81) | 1.34 (0.98, 1.84) | 1.33 (0.98, 1.81) | 0.283 | 1.29 (0.92, 1.81) | 1.34 (0.97, 1.85) | 1.35 (0.99, 1.84) | 0.178 |
| Q2: >17.46 - ≤30.14 | 1.08 (0.87, 1.35) | 1.06 (0.86, 1.31) | 1.09 (0.88, 1.35) | 0.556 | 1.08 (0.86, 1.35) | 1.01 (0.82, 1.26) | 1.07 (0.86, 1.32) | 0.719 |
| Q3: >30.14 - ≤49.24 | 1.08 (0.92, 1.27) | 1.12 (0.96, 1.32) | 1.14 (0.95, 1.37) | 0.148 | 1.03 (0.87, 1.21) | 1.11 (0.94, 1.31) | 1.15 (0.95, 1.38) | 0.091 |
| Q4: >49.24 | 1.10 (0.96, 1.25) | 1.01 (0.86, 1.19) | 0.96 (0.71, 1.30) | 0.815 | 1.08 (0.94, 1.22) | 1 (0.85, 1.17) | 0.97 (0.72, 1.32) | 0.915 |
| P for interaction[b] | 0.789 | | | | 0.983 | | | |
| Natural environment percentage, buffer 1000m | | | | | | | | |
| Q1: ≤19.98 | 1.53 (1.16, 2.02) | 1.43 (1.10, 1.86) | 1.72 (1.36, 2.11) | <0.001 | 1.58 (1.18, 2.11) | 1.49 (1.14, 1.95) | 1.85 (1.44, 2.37) | <0.001 |
| Q2: >19.98 - ≤37.82 | 1.46 (1.20, 1.78) | 1.43 (1.19, 1.73) | 1.34 (1.11, 1.63) | 0.075 | 1.53 (1.25, 1.88) | 1.5 (1.24, 1.82) | 1.46 (1.2, 1.78) | 0.012 |
| Q3: >37.82 - ≤59.71 | 1.05 (0.91, 1.21) | 1.14 (0.98, 1.31) | 0.97 (0.79, 1.20) | 0.584 | 1.04 (0.9, 1.2) | 1.14 (0.99, 1.32) | 1 (0.81, 1.23) | 0.441 |
| Q4: >59.71 | 1.14 (1.01, 1.29) | 1.07 (0.91, 1.25) | 1.02 (0.68, 1.54) | 0.198 | 1.13 (1, 1.28) | 1.08 (0.93, 1.27) | 1.05 (0.7, 1.58) | 0.160 |
| P for interaction[b] | 0.136 | | | | 0.058 | | | |
| Natural environment percentage, buffer 300m | | | | | | | | |
| Q1: ≤6.47 | 1.36 (1.07, 1.72) | 1.46 (1.17, 1.82) | 1.46 (1.18, 1.79) | 0.003 | 1.44 (1.13, 1.85) | 1.58 (1.26, 1.99) | 1.61 (1.3, 2) | <0.001 |
| Q2: >6.47 - ≤19.64 | 1.39 (1.15, 1.67) | 1.19 (0.99, 1.43) | 1.35 (1.13, 1.62) | 0.017 | 1.42 (1.17, 1.72) | 1.24 (1.03, 1.49) | 1.42 (1.18, 1.71) | 0.004 |
| Q3: >19.64 - ≤40.40 | 1.05 (0.91, 1.22) | 1.12 (0.97, 1.30) | 1.01 (0.85, 1.20) | 0.672 | 1.06 (0.91, 1.23) | 1.12 (0.97, 1.31) | 1.05 (0.88, 1.25) | 0.408 |
| Q4: >40.40 | 1.16 (1.03, 1.31) | 1.18 (1.01, 1.37) | 1.19 (0.93, 1.53) | 0.013 | 1.14 (1.01, 1.29) | 1.16 (1, 1.35) | 1.21 (0.94, 1.56) | 0.016 |
| P for interaction[b] | 0.725 | | | | 0.318 | | | |
| NDVI mean, buffer 500m | | | | | | | | |
| Q1: ≤0.01 | 0.88 (0.72, 1.07) | 0.92 (0.75, 1.12) | 0.86 (0.68, 1.08) | 0.276 | 0.85 (0.69, 1.04) | 0.91 (0.74, 1.11) | 0.85 (0.67, 1.08) | 0.293 |
| Q2: >0.01 - ≤0.11 | 1.02 (0.81, 1.27) | 1.00 (0.8, 1.24) | 1.07 (0.87, 1.31) | 0.498 | 1.03 (0.82, 1.29) | 1.03 (0.83, 1.29) | 1.12 (0.9, 1.38) | 0.248 |
| Q3: >0.11 - ≤0.23 | 1.12 (0.91, 1.36) | 0.93 (0.76, 1.14) | 1.02 (0.83, 1.25) | 0.705 | 1.11 (0.91, 1.36) | 0.93 (0.76, 1.15) | 1.02 (0.83, 1.25) | 0.732 |
| Q4: >0.23 | 1.10 (0.84, 1.45) | 1.20 (0.92, 1.55) | 1.13 (0.90, 1.41) | 0.445 | 1.08 (0.82, 1.42) | 1.12 (0.86, 1.46) | 1.12 (0.89, 1.4) | 0.393 |
| P for interaction[b] | 0.732 | | | | 0.899 | | | |

**Abbreviations**: NDVI - normalized difference vegetation index; $PM_{10}$ - particulate matter≤10 μm in diameter; Q - quartile

[a]Risk estimates adjusted for age, body mass index, race, age at menopause, age at menarche, parity/age at first birth, postmenopausal hormone use, family history of breast cancer, alcohol consumption, and smoking; [b]P for interaction between air pollutant measure and greenness measure, using the respective medians within each of the exposure quartiles; [c]P for trend using the median air pollutant level within each quartile of greenness measure
