# Supplementary material for: Greenness and its interaction with air pollution in relation to postmenopausal breast cancer risk in UK Biobank
Source: PLoS One. 2025 Nov 12;20(11):e0334744. doi: 10.1371/journal.pone.0334744 (PMC12611134; doi:10.1371/journal.pone.0334744)
Supplement: S5 Table — (PDF) [file pone.0334744.s005.pdf]

S5 Table. Association of quartiles of 2010 PM_{10} with invasive breast cancer risk, by the quartiles of the greenness measures, without and with 2-year air pollution exposure lag (hazard ratios and 95% confidence intervals)[a]

Greenness measure	Without air pollution exposure lag				With 2-year air pollution exposure lag			
	2010 PM_{10} quartile (μg/m^3)				2010 PM_{10} quartile (μg/m^3)			
	2nd (>15.22-≤16.01)	3rd (>16.01-≤16.98)	4th (>16.98)	P for trend[c]	2nd (>15.22-≤16.01)	3rd (>16.01-≤16.98)	4th (>16.98)	P for trend[c]
Greenspace percentage, buffer 1000m.								
Q1: ≤27.94	0.85 (0.63, 1.15)	0.94 (0.70, 1.25)	0.94 (0.71, 1.26)	0.652	0.85 (0.61, 1.18)	0.92 (0.67, 1.26)	0.95 (0.69, 1.30)	0.553
Q2: >27.94 - ≤42.54	0.91 (0.75, 1.11)	0.94 (0.77, 1.14)	0.86 (0.70, 1.05)	0.168	0.89 (0.71, 1.12)	0.98 (0.79, 1.22)	0.86 (0.69, 1.09)	0.294
Q3: >42.54 - ≤60.91	0.90 (0.77, 1.06)	0.92 (0.78, 1.1)	0.94 (0.79, 1.12)	0.529	0.86 (0.72, 1.03)	0.98 (0.81, 1.18)	0.86 (0.71, 1.05)	0.234
Q4: >60.91	1.06 (0.90, 1.24)	0.96 (0.78, 1.19)	1.11 (0.95, 1.31)	0.266	1.04 (0.87, 1.24)	0.88 (0.70, 1.12)	1.14 (0.96, 1.37)	0.268
P for interaction[b]	0.270				0.906			
Greenspace percentage, buffer 300m								
Q1: ≤17.46	0.75 (0.54, 1.05)	0.88 (0.64, 1.22)	0.81 (0.58, 1.13)	0.802	0.70 (0.48, 1.00)	0.86 (0.61, 1.23)	0.81 (0.57, 1.16)	0.760
Q2: >17.46 - ≤30.14	1.11 (0.88, 1.39)	1.05 (0.84, 1.32)	1.09 (0.87, 1.38)	0.662	0.99 (0.78, 1.27)	0.98 (0.77, 1.25)	0.99 (0.77, 1.28)	0.984
Q3: >30.14 - ≤49.24	0.79 (1.08, 1.03)	0.77 (1.08, 1.05)	0.82 (1.14, 0.97)	0.660	0.90 (0.76, 1.08)	0.97 (0.80, 1.16)	0.95 (0.79, 1.15)	0.726
Q4: >49.24	1.00 (0.84, 1.19)	1.00 (0.81, 1.24)	1.03 (0.87, 1.21)	0.785	1.00 (0.83, 1.22)	0.94 (0.73, 1.02)	0.98 (0.81, 1.18)	0.731
P for interaction[b]	0.491				0.548			
Natural environment percentage, buffer 1000m								
Q1: ≤19.98	0.55 (1.01, 1.03)	0.62 (1.08, 1.03)	0.62 (1.10, 1.11)	0.935	0.71 (0.51, 1.00)	0.81 (0.59, 1.10)	0.86 (0.63, 1.17)	0.423
Q2: >19.98 - ≤37.82	1.00 (0.82, 1.23)	1.05 (0.86, 1.29)	0.96 (0.78, 1.19)	0.597	1.05 (0.83, 1.33)	1.15 (0.91, 1.45)	0.99 (0.78, 1.26)	0.719
Q3: >37.82 - ≤59.71	0.86 (0.73, 1.00)	0.91 (0.77, 1.07)	0.85 (0.72, 1.01)	0.091	0.80 (0.68, 0.95)	0.93 (0.78, 1.12)	0.76 (0.63, 0.92)	0.016
Q4: >59.71	1.07 (0.92, 1.26)	0.91 (0.74, 1.12)	1.12 (0.96, 1.32)	0.261	1.06 (0.89, 1.26)	0.86 (0.68, 1.09)	1.15 (0.96, 1.37)	0.258
P for interaction[b]	0.344				0.941			
Natural environment percentage, buffer 300m								
Q1: ≤6.47	0.65 (0.48, 0.89)	0.76 (0.56, 1.01)	0.70 (0.52, 0.94)	0.394	0.59 (0.43, 0.83)	0.70 (0.51, 0.96)	0.67 (0.49, 0.92)	0.658
Q2: >6.47 - ≤19.64	0.95 (0.76, 1.20)	0.98 (0.78, 1.23)	0.96 (0.76, 1.22)	0.898	0.83 (0.64, 1.06)	0.95 (0.74, 1.22)	0.91 (0.71, 1.17)	0.914
Q3: >19.64 - ≤40.40	1.02 (0.87, 1.19)	0.97 (0.82, 1.15)	1.00 (0.84, 1.18)	0.886	1.04 (0.87, 1.24)	1.00 (0.83, 1.21)	0.99 (0.81, 1.20)	0.820
Q4: >40.40	1.00 (0.84, 1.18)	0.96 (0.78, 1.19)	1.06 (0.90, 1.24)	0.613	1.02 (0.84, 1.23)	0.99 (0.79, 1.25)	1.00 (0.83, 1.20)	0.980
P for interaction[b]	0.445				0.861			
NDVI mean, buffer 500m								
Q1: ≤0.01	0.81 (0.65, 1.00)	0.89 (0.73, 1.09)	0.75 (0.60, 0.95)	0.030	0.73 (0.57, 0.92)	0.88 (0.71, 1.10)	0.70 (0.54, 0.91)	0.023
Q2: >0.01 - ≤0.11	1.02 (0.83, 1.25)	1.25 (1.03, 1.53)	1.15 (0.93, 1.43)	0.095	1.04 (0.83, 1.31)	1.24 (0.99, 1.54)	1.12 (0.88, 1.43)	0.224
Q3: >0.11 - ≤0.23	0.99 (0.82, 1.21)	0.90 (0.74, 1.09)	0.95 (0.78, 1.16)	0.511	0.97 (0.78, 1.20)	0.88 (0.71, 1.09)	0.96 (0.77, 1.19)	0.625
Q4: >0.23	1.03 (0.82, 1.30)	0.97 (0.79, 1.20)	1.05 (0.87, 1.27)	0.627	1.00 (0.77, 1.29)	0.95 (0.75, 1.20)	1.02 (0.82, 1.25)	0.848
P for interaction[b]	0.376				0.274			

Abbreviations: NDVI - normalized difference vegetation index; PM_{10} - particulate matter≤10 μm in diameter; Q - quartiles

[a]Risk estimates adjusted for age, body mass index, race, age at menopause, age at menarche, parity/age at first birth, postmenopausal hormone use, family history of breast cancer, alcohol consumption, and smoking; [b]P for interaction between air pollutant measure and greenness measure, using the respective medians within each of the exposure quartiles; [c]P for trend using the median air pollutant level within each quartile of greenness measure
