# Supplementary material for: Greenness and its interaction with air pollution in relation to postmenopausal breast cancer risk in UK Biobank
Source: PLoS One. 2025 Nov 12;20(11):e0334744. doi: 10.1371/journal.pone.0334744 (PMC12611134; doi:10.1371/journal.pone.0334744)
Supplement: S6 Table — (PDF) [file pone.0334744.s006.pdf]

S6 Table. Association of quartiles of cumulative average PM_{10} with breast cancer risk, by the quartiles of the greenness measures, without and with 2-year air pollution exposure lag (hazard ratios and 95% confidence intervals)[a]

Greenness measure	Without air pollution exposure lag				With 2-year air pollution exposure lag			
	Cumulative average PM_{10} quartile (μg/m^3)				Cumulative average PM_{10} quartile (μg/m^3)			
	2nd (>17.92-≤19.04)	3rd (>19.04-≤20.25)	4th (>20.25)	P for trend[c]	2nd (>17.92-≤19.04)	3rd (>19.04-≤20.25)	4th (>20.25)	P for trend[c]
Greenspace percentage, buffer 1000m.								
Q1: ≤27.94	0.81 (0.55, 1.21)	0.82 (0.56, 1.19)	1.07 (0.74, 1.55)	<0.001	0.77 (0.5, 1.17)	0.75 (0.5, 1.11)	0.96 (0.65, 1.42)	0.009
Q2: >27.94 - ≤42.54	1.27 (0.99, 1.63)	1.18 (0.92, 1.50)	1.54 (1.21, 1.97)	<0.001	1.31 (0.98, 1.74)	1.25 (0.94, 1.66)	1.56 (1.18, 2.07)	<0.001
Q3: >42.54 - ≤60.91	0.94 (0.81, 1.10)	1.12 (0.95, 1.32)	1.46 (1.22, 1.75)	<0.001	0.92 (0.77, 1.10)	1.12 (0.94, 1.34)	1.34 (1.09, 1.65)	0.001
Q4: >60.91	1.14 (0.99, 1.31)	1.23 (1.03, 1.47)	1.95 (1.60, 2.39)	<0.001	1.12 (0.96, 1.31)	1.25 (1.00, 1.52)	1.89 (1.50, 2.37)	<0.001
P for interaction[b]	0.052				0.387			
Greenspace percentage, buffer 300m								
Q1: ≤17.46	1.23 (0.86, 1.75)	1.27 (0.90, 1.80)	1.49 (1.06, 2.09)	0.001	1.07 (0.73, 1.57)	1.14 (0.79, 1.64)	1.35 (0.95, 1.92)	0.003
Q2: >17.46 - ≤30.14	1.11 (0.88, 1.40)	1.01 (0.80, 1.27)	1.43 (1.14, 1.79)	<0.001	1.06 (0.82, 1.36)	0.97 (0.75, 1.25)	1.27 (0.99, 1.62)	0.011
Q3: >30.14 - ≤49.24	1.01 (0.86, 1.19)	1.19 (1.01, 1.40)	1.48 (1.25, 1.76)	<0.001	1.00 (0.83, 1.20)	1.28 (1.06, 1.53)	1.44 (1.19, 1.75)	<0.001
Q4: >49.24	1.10 (0.95, 1.26)	1.11 (0.93, 1.34)	1.84 (1.52, 2.22)	<0.001	1.12 (0.96, 1.31)	1.04 (0.84, 1.28)	1.68 (1.35, 2.09)	0.001
P for interaction[b]	0.067				0.530			
Natural environment percentage, buffer 1000m								
Q1: ≤19.98	0.74 (0.52, 1.06)	0.70 (0.50, 0.99)	0.98 (0.71, 1.37)	<0.001	0.68 (0.46, 1.01)	0.67 (0.46, 0.96)	0.9 (0.63, 1.29)	<0.001
Q2: >19.98 - ≤37.82	1.24 (0.99, 1.55)	1.26 (1.01, 1.57)	1.67 (1.34, 2.08)	<0.001	1.23 (0.96, 1.58)	1.29 (1.01, 1.65)	1.63 (1.28, 2.09)	<0.001
Q3: >37.82 - ≤59.71	1.03 (0.89, 1.19)	1.19 (1.02, 1.39)	1.45 (1.22, 1.73)	<0.001	1.00 (0.85, 1.18)	1.17 (0.99, 1.39)	1.26 (1.03, 1.54)	<0.001
Q4: >59.71	1.11 (0.97, 1.26)	1.22 (1.03, 1.45)	2.01 (1.66, 2.42)	<0.001	1.12 (0.96, 1.3)	1.21 (1.00, 1.47)	1.98 (1.61, 2.45)	<0.001
P for interaction[b]	0.151				0.571			
Natural environment percentage, buffer 300m								
Q1: ≤6.47	1.29 (0.94, 1.76)	1.25 (0.92, 1.70)	1.53 (1.14, 2.06)	<0.001	1.05 (0.75, 1.46)	1.08 (0.79, 1.48)	1.32 (0.97, 1.79)	0.0013
Q2: >6.47 - ≤19.64	1.12 (0.91, 1.37)	1.05 (0.86, 1.29)	1.53 (1.25, 1.87)	<0.001	1.10 (0.87, 1.39)	1.09 (0.87, 1.36)	1.46 (1.17, 1.83)	<0.001
Q3: >19.64 - ≤40.40	0.98 (0.84, 1.14)	1.13 (0.97, 1.32)	1.39 (1.18, 1.64)	<0.001	1.00 (0.85, 1.19)	1.16 (0.98, 1.38)	1.28 (1.06, 1.55)	0.003
Q4: >40.40	1.12 (0.98, 1.28)	1.23 (1.04, 1.45)	1.96 (1.64, 2.34)	<0.001	1.14 (0.98, 1.32)	1.20 (1.00, 1.44)	1.87 (1.53, 2.29)	<0.001
P for interaction[b]	0.125				0.347			
NDVI mean, buffer 500m								
Q1: ≤0.01	0.84 (0.69, 1.03)	0.93 (0.76, 1.15)	1.29 (1.05, 1.58)	0.004	0.77 (0.62, 0.96)	0.93 (0.74, 1.16)	1.10 (0.87, 1.39)	0.189
Q2: >0.01 - ≤0.11	1.18 (0.95, 1.48)	1.31 (1.06, 1.61)	1.40 (1.14, 1.73)	0.001	1.34 (1.04, 1.73)	1.42 (1.12, 1.81)	1.47 (1.15, 1.87)	0.004
Q3: >0.11 - ≤0.23	0.98 (0.81, 1.19)	0.87 (0.71, 1.07)	1.16 (0.96, 1.41)	0.163	0.91 (0.73, 1.13)	0.84 (0.67, 1.05)	1.19 (0.96, 1.46)	0.105
Q4: >0.23	0.92 (0.70, 1.21)	1.00 (0.78, 1.28)	1.17 (0.96, 1.42)	0.031	0.93 (0.69, 1.26)	0.95 (0.73, 1.25)	1.11 (0.90, 1.38)	0.154
P for interaction[b]	0.111				0.024			

Abbreviations: NDVI - normalized difference vegetation index; PM_{10} - particulate matter≤10 μm in diameter; Q - quartiles
[a]Risk estimates adjusted for age, body mass index, race, age at menopause, age at menarche, parity/age at first birth, postmenopausal hormone use, family history of breast cancer, alcohol consumption, and smoking; [b]P for interaction between air pollutant measure and greenness measure, using the respective medians within each of the exposure quartiles; [c]P for trend using the median air pollutant level
